# A SPR biosensor based on signal amplification using antibody-QD conjugates for quantitative determination of multiple tumor markers

**DOI:** 10.1038/srep33140

**Published:** 2016-09-12

**Authors:** Huan Wang, Xiaomei Wang, Jue Wang, Weiling Fu, Chunyan Yao

**Affiliations:** 1 Department of Laboratory Medicine, Southwest Hospital, the Third Military Medical University, Chongqing 400038, China; 2 Chongqing Communication Institute, Chongqing 400035, China; 3 Department of Geriatrics, Southwest Hospital, the Third Military Medical University, Chongqing 400038, China

## Abstract

The detection of tumor markers is very important in early cancer diagnosis; however, tumor markers are usually present at very low concentrations, especially in the early stages of tumor development. Surface plasmon resonance (SPR) is widely used to detect biomolecular interactions; it has inherent advantages of being high-throughput, real-time, and label-free technique. However, its sensitivity needs essential improvement for practical applications. In this study, we developed a signal amplification strategy using antibody-quantum dot (QD) conjugates for the sensitive and quantitative detection of α-fetoprotein (AFP), carcinoembryonic antigen (CEA) and cytokeratin fragment 21-1 (CYFRA 21-1) in clinical samples. The use of a dual signal amplification strategy using AuNP-antibody and antibody-QD conjugates increased the signal amplification by 50-folds. The constructed SPR biosensor showed a detection limit as low as 0.1 ng/mL for AFP, CEA, and CYFRA 21-1. Moreover, the results obtained using this SPR biosensor were consistent with those obtained using the electrochemiluminescence method. Thus, the constructed SPR biosensor provides a highly sensitive and specific approach for the detection of tumor markers. This SPR biosensor can be expected to be readily applied for the detection of other tumor markers and can offer a potentially powerful solution for tumor screening.

Cancer is a broad group of complex diseases that greatly affects human health worldwide. It is also the leading cause of death and accounted for 8.2 million deaths in 2012, according to the World Cancer Report 2014 published by the WHO. Tumor markers are molecules that are abnormally expressed in malignant and benign tumors^1^,^2^. Thus, the detection of tumor markers is of immense significance in early cancer diagnosis, evaluation the extent of the disease, and monitoring of therapy response^3^,^4^. Several groups have proposed methods for the sensitive quantification of tumor markers using techniques such as electrochemistry^5^,^6^, fluorescence^7^,^8^,^9^, electrochemiluminescence^10^,^11^, and biosensors^12^,^13^,^14^. Electrochemiluminescence is a conventional method used for detecting tumor markers; however, it requires expensive instruments and reagents, bulky platforms and has low sensitivity for biological fluids^15^,^16^. Recently, various biosensors have been reported for the multianalysis of tumor markers. Cheng *et al.* developed a field effect transistor (FET) biosensor that allows the label-free detection of CYFRA 21-1 and neuron-specific enolase (NSE), two useful tumor markers for lung cancer^17^. A label-free immunoassay strategy based on modified mesoporous silica was also proposed by Lin *et al.* for the simultaneous detection of CEA and AFP^18^.

Since many tumor markers are present at very low concentrations in the serum, many researchers have proposed reducing the limit of detection (LOD) for tumor markers to enable cancer diagnosis at a very early stage^19^,^20^. Surface plasmon resonance (SPR) is a highly sensitive method for the label-free detection of biomolecular interactions^21^,^22^. Considering the low concentrations of tumor markers, signal amplification would be necessary if a SPR biosensor was to be used. Different approaches have been used for signal amplification in SPR assays, such as functionalized nanoparticles^23^,^24^, isothermal amplification^25^,^26^, and quantum dots^27^,^28^. Martinez-Perdiguero *et al.* used amplification steps with high affinity biotinylated antibodies and streptavidin-functionalized nanoparticles, which greatly enhanced the detection signal^24^. In Chuang’s study, a SPR-based loop-mediated isothermal amplification (LAMP) sensing system was constructed, and the hepatitis B virus (HBV) target was detected with a detection limit of 2 fg/mL^25^. In exchange for the loss of the label-free format, amplification steps can provide the necessary sensitivity to detect analytes present at low concentrations. Quantum dots (QDs) have attracted much attention in biological imaging^29^,^30^,^31^, DNA detection^32^,^33^, multiplexing beads^34^, and as efficient donors in fluorescence resonance energy transfer (FRET) mechanisms^35^,^36^. Due to their unique optical and electrical properties such as broad excitation region, narrow emission area, tuneable optical properties, strong luminescence, and excellent photostability, QDs are used as a robust reporter to develop sensitive biosensors with multianalyte quantification capability^37^,^38^,^39^. In this regard, some researchers have been working on combination of QDs and SPR biosensors to achieve high sensitivity and low LOD; some such studies have also been focused on the detection of tumor markers^40^,^41^. An ultrasensitive QD-based SPR imaging biosensor was studied by Malic *et al.* where the detection limit for prostate specific antigen (PSA) was reduced to 100 pg/mL after amplification^42^.

Early screening is very useful for early cancer diagnosis. Quantitative analysis of a set of tumor markers is useful in cancer screening. Liver cancer, lung cancer, and colorectal cancer are the most commonly diagnosed malignancies in China^43^. Among them, liver cancer and lung cancer are the most common causes of cancer-related deaths. AFP is an important tumor marker that is closely associated with the diagnosis of hepatocellular carcinoma and other chronic liver diseases^40^. CYFRA 21-1 is a useful tumor marker for lung cancer^44^. Meanwhile, CEA is a known tumor marker that is used to monitor colorectal cancer^45^,^46^. Since the totally morbidity associated with liver cancer, lung cancer, and colorectal cancer accounts for 30% of the morbidity associated with all types of cancer in China^43^, we chose these three tumor markers as targets with an aim to provide a facile biosensor with diagnostic and prognostic significance for cancer screening. In the present study, we developed a new method using a multi-channel SPR biosensor to accurately and simultaneously detect AFP, CEA, and CYFRA 21-1. A dual signal amplification strategy using AuNP-antibody conjugates and antibody-QD conjugates was designed to improve the sensitivity of detection. The sensitivity, specificity, and selectivity of the SPR biosensor were also evaluated. To the best of our knowledge, this is the first report on the simultaneous detection of AFP, CEA, and CYFRA 21-1 using an antibody-QD conjugate amplification-based SPR biosensor.

## Results

### Design strategy for the SPR biosensor

**Figure 1:**
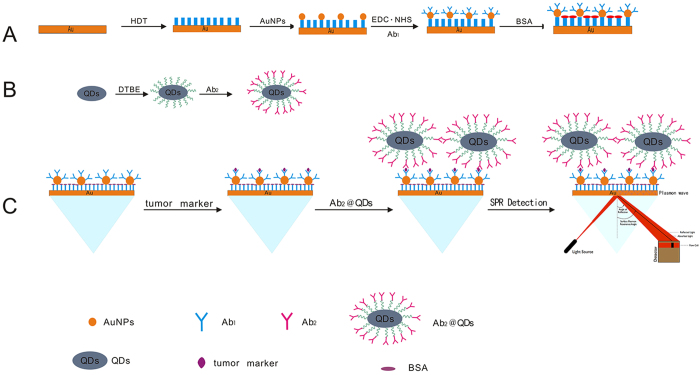
Schematic diagram of the detection procedure using the SPR biosensor. (**A**) Coating of an SPR chip with AuNP@Ab_1_ conjugates. (**B**) Preparation of Ab_2_@QD conjugates. (**C**) Sample was flowed along the sensor chip coated with AuNP@Ab_1_ conjugates to capture the target, followed by running the Ab_2_@QD conjugates to amplify the signal, which could be detected by the SPR biosensor.

SPR is an optical non-destructive method that can detect very small changes in the refractive index (RI) of a typically gold sensor surface. With adequate surface biofunctionalization, the binding of a given analyte results in a change in the RI, which in turn is measured as a change in the SPR angle. Hexanedithiol (HDT) was used as a chemical linker for the formation of the AuNP monolayer to produce an active interface on the chip surface in order to improve the sensitivity of detection. This yielded a good dispersion of the AuNPs and an increased surface area for site-specific binding. The AuNP@Ab_1_ conjugates were immobilized on the surface of the chip through chemical modification. When the target and Ab_2_@QD conjugates captured by the AuNP@Ab_1_ conjugates, the corresponding refractive angle changes caused by the immunoreaction were recorded in a real-time manner and these changes were proportional to the mass of the target. Due to the mass enhancement of the QDs, the changes in the signal of the SPR biosensor increased, and the amount of target could be determined. The basic principle of the proposed antibody-QD amplification-based SPR method is presented schematically in Fig. 1.

### Optimization of experimental parameters

In order to establish the optimal conditions for the detection, we varied the concentrations of Ab_1_ and the ratio of Ab_2_ to QDs. The amount of Ab_1_ immobilized on the chip surface was a key parameter in the immunoassay format. The concentration of Ab_1_ is an important factor affecting the detection limit because a low concentration on the chip surface leads to a lower upper limit of detection, whereas any redundant antibody will significantly increase steric hindrance on the immunoassay. As shown in Supplementary Fig. 1A, the change in the SPR signal increased with increasing concentration of Ab_1_ and reached a peak at 0.5 mg/mL for anti-AFP_1_, 0.6 mg/mL for anti-CEA_1_ and 0.65 mg/mL for anti-CYFRA 21-1_1_, after this point, the change in the SPR signal decreased, which showed that the chip surface gradually became saturated. Therefore, 0.5 mg/mL, 0.6 mg/mL, and 0.65 mg/mL were selected as the optimal concentrations for anti-AFP_1_, anti-CEA_1_, and anti-CYFRA 21-1_1_, respectively. Next, we investigated the optimal ratio of Ab_2_ to QDs for detection using SPR. Different ratios of Ab_2_/QDs were tested (5:1, 10:1, 20:1, 50:1, and 100:1). Supplementary Fig. 1B shows the change in the SPR signal under different Ab_2_/QDs ratios. The change in the SPR signal increased with increasing Ab_2_/QDs ratio and reached its maximum at a 20:1 ratio of Ab_2_/QDs. Beyond that ratio, the increase trend weakened gradually, which may have been because the QD surface became saturated. Based on these results, 20:1 was chosen as the optimal ratio of Ab_2_/QDs for the following experiments.

### Evaluation of the conjugation of Ab_2_ and QDs

In order to confirm the binding of the QDs and Ab_2_, the fluorescence characteristics of the QDs before and after coupling with Ab_2_ were measured. As shown in Supplementary Fig. 2, the fluorescence intensity of anti-AFP_2_@QDs, anti-CEA_2_@QDs, and anti-CYFRA 21-1_2_@QDs became brighter in comparison with that of the un-conjugated QDs (for the same concentration of QDs). The probable reason for this is that the conjugated antibody in the Ab_2_@QD conjugates enhanced the fluorescence intensity of the solution, which also proved that the antibody had successfully combined with the QDs.

### Evaluation of the signal amplification function of Ab_2_@QD conjugates

**Figure 2:**
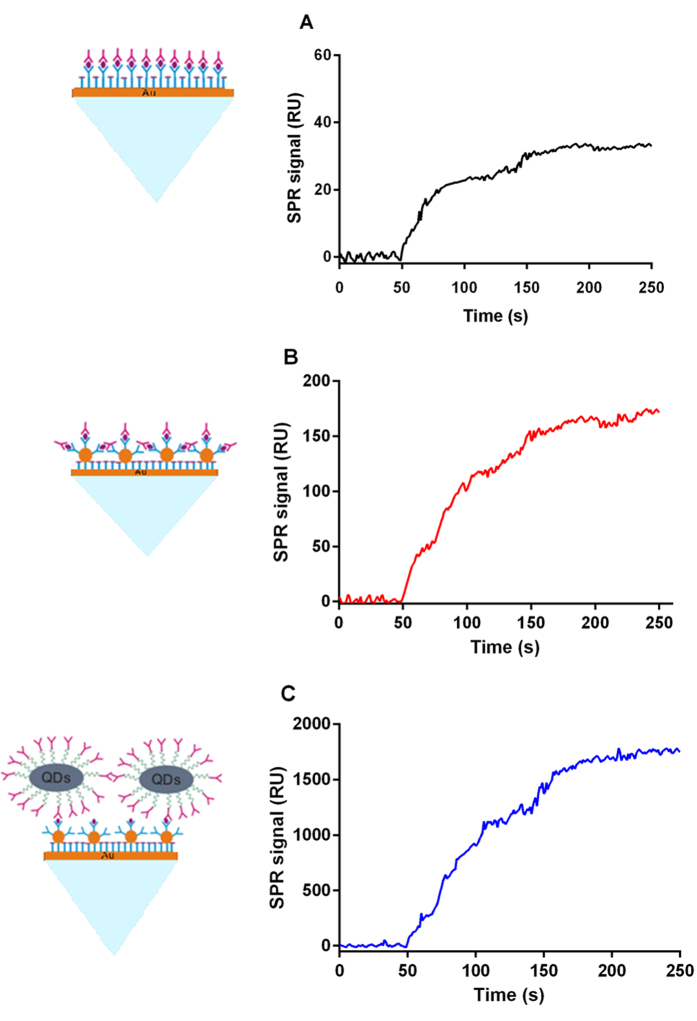
Comparison of different signal amplification methods. (**A**) Direct assay for AFP. (**B**) Detection with signal amplification using AuNP@Ab_1_ conjugates. (**C**) Detection with dual signal amplification using AuNP@Ab_1_ and Ab_2_@QD conjugates. The concentration of the AFP and anti-AFP_2_ was 100 ng/mL and 0.1 mg/mL, respectively.

To evaluate the signal amplification function of the antibody-QD conjugates, the fabricated SPR biosensor was used for the detection of AFP. First, the chip, which was coated with AuNP@anti-AFP_1_ conjugates or only anti-AFP_1_, was exposed to AFP. After the reaction, the surface of the chip was flushed with buffer to wash away the non-specifically bound AFP, while the bound AFP was further detected by the addition of either free anti-AFP_2_ or anti-AFP_2_@QD conjugates. Figure 2A shows the direct binding of AFP, and Fig. 2B shows the results of AFP detection by AuNP@anti-AFP_1_ conjugates and free anti-AFP_2_. AFP detection with AuNP@anti-AFP_1_ and anti-AFP_2_@QD conjugates is also shown in Fig. 2C. All the detections were carried out under the same conditions. The anti-AFP_2_@QD conjugates clearly generated a much larger signal than the free anti-AFP_2_, as seen by comparing Fig. 2B,C. A 10.2-fold greater amplification was observed with use of the anti-AFP_2_@QD conjugates in this format.

### Sensitivity and precision of the detection using the SPR biosensor

**Figure 3:**
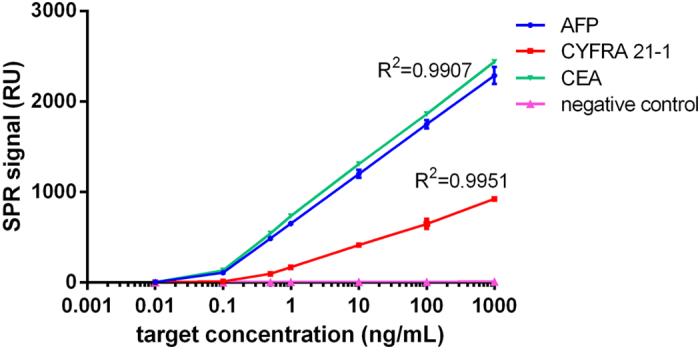
Standard curves for the quantitative detection of AFP, CEA, and CYFRA 21-1 using the SPR biosensor.

The response of the AuNP-antibody-coated chips to the tumor markers was evaluated. The relation between the concentrations of the targets and the SPR signals is shown in Fig. 3. Addition of antibody-QD conjugates provided a striking signal enhancement, which translated to an improved LOD. In addition, the response intensities were logarithmically proportional to the target concentration in the range from 10^−1^ to 10^3 ^ng/mL, and the equations for the linear regressions were RU = 654.9 + 546.0 × log C for AFP, RU = 718.2 + 569.8 × log C for CEA, and RU = 169.2 + 243.1 × log C for CYFRA 21-1 (Fig. 3). The LOD was determined as the concentration of the target to be detected using the SPR biosensor corresponding to the blank control signal plus three times of the standard deviation. PBS buffer instead of the target was used as the blank control. The LOD for AFP, CEA, and CYFRA 21-1 was 0.1 ng/mL.

### Specificity of detection using the SPR biosensor

**Figure 4:**
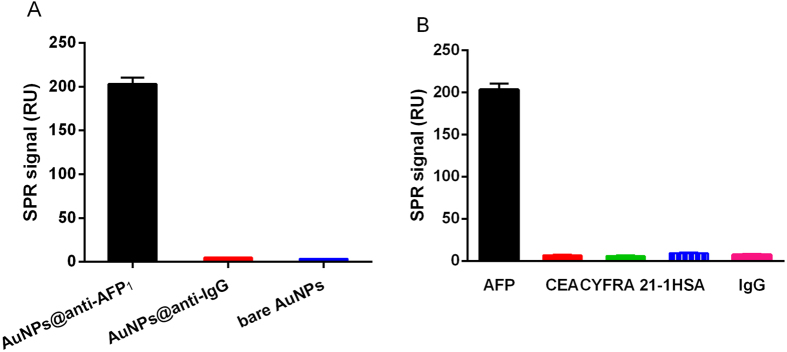
Assessment of specificity and selectivity of the SPR biosensor. (**A**) Detection of AFP on AuNP@anti-AFP_1_-coated chips, AuNP@anti-IgG-coated chips, and bare AuNP surface. The concentration of AFP was 100 ng/mL. (**B**) Cross-reaction of CEA, CYFRA 21-1, HSA, and IgG was detected using AuNP@anti-AFP_1_ conjugate-coated chips. The concentration of AFP was 100 ng/mL, and that of each of the other targets was 200 ng/mL.

The specificity of detection using the SPR biosensor was determined by comparing the changes in the signals of the SPR chip coated with AuNP@anti-AFP_1_ conjugates with that of the chip coated with AuNP@anti-IgG and the bare AuNP chips without antibody coating. The results showed that the changes in the SPR angle were minimally affected by treatment of the bare chips and anti-IgG-coated chips with AFP (Fig. 4A). We further compared the changes in the SPR signal caused by the other two kinds of tumor markers with cross-reactions. While using AuNP@anti-AFP_1_-coated chips with CEA and CYFRA 21-1 as the targets, the changes in the SPR angle were small, and there was no significant difference between the changes in the SPR signal for the miss-targets (Fig. 4B). Thus, the cross-reactivity and nonspecific binding of detection were negligible, suggesting that the three tumor markers could be assayed individually in a single run without interference.

The selectivity of the SPR biosensor was further explored using HSA and IgG as the targets, both of which are present naturally in human serum samples. No significant signal changes were observed when HSA or IgG were applied to the AuNP@anti-AFP_1_ conjugate-coated chips, as presented in Fig. 4B. These findings show that the SPR biosensor was selective for AFP in a manner that was not affected by the presence of other contaminating proteins in the serum samples.

### Application of the SPR biosensor for clinical samples

To evaluate the reliability and application potential of the proposed multianalyte SPR biosensor, a series of samples were prepared by spiking AFP, CEA and CYFRA 21-1 at different concentrations to the human serum samples. As shown in Table 1, the recovery rates were in an acceptable range from 95% to 110% for AFP, from 90% to 115% for CEA, and from 95% to 105% for CYFRA 21-1, indicating that the proposed biosensor had good accuracy in the sample matrix.

Finally, parallel detection of three tumor markers in serum samples was carried out to evaluate the accuracy of the proposed method. Ten clinical samples were analyzed using the SPR biosensor and the electrochemiluminescence method. The relative errors between the two methods were less than 10%, showing an acceptable accuracy (Table 2). The assay results for the clinical serum samples using the SPR biosensor were in agreement with those obtained using the electrochemiluminescence method.

## Discussion

Although many treatment modalities and drugs have been developed over the past several decades, the 5-year survival rate for cancer remains low. Early diagnoses of cancer can effectively reduce cancer-related mortality. The serum levels of tumor markers carry prognostic and predictive information about the risk of incidence, recurrence, and death associated with cancer. Numerous studies on biomolecular interactions have been conducted with SPR, including DNA, RNA, proteins, and peptides. SPR has also been used for the analysis of tumor markers^47^,^48^. For example, a proof of concept screening for monoclonal immunoglobulin as a leukemia tumor marker was conducted by Maisonneuve *et al.* using a SPR biosensing platform^47^. Chou *et al.* developed a self-assembled SPR apparatus for the detection of ferritin^49^.

In this study, the high affinity between the conjugated AuNP@Ab_1_ and the targets directed the Ab_2_@QD conjugates to bind onto the sensing surface, resulting in effective signal enhancement. The reaction resulted in obvious changes in the SPR signal, thus offering a significant amplification for detection. The amplification factor of the AuNP@Ab_1_ conjugates with respect to the initial sample signal was 5.1. Sequentially, Ab_2_@QD conjugates further enhanced this signal by a factor of 10.2. The additive effect of the two amplification steps resulted in an effective signal amplification factor of about 50, indicating that the introduction of antibody-QD conjugates was effective in improving the sensitivity of detection using the SPR biosensor. The results also showed that the prepared SPR biosensor had a relatively larger linear range and lower LOD compared to that of the other determination method used for tumor marker detection (Supplementary Table 1). Considering the typical concentrations of AFP (normal range: <20 ng/mL, approximately 700 ng/mL during disease state), CEA, and CYFRA 21-1 (up to several hundred ng/mL during disease state) in the plasma^48^, our reported strategy allows the SPR biosensor to fully meet the clinical requirements. Specific binding of the high-affinity immunoassay and the amplification using the antibody-QD conjugates not only amplify the initial signal, but also improve the specificity and selectivity of the assay. The results indicated that the proposed SPR method could distinguish between different tumor markers due to the high specificity of the immune reaction. Thus, the constructed antibody-QD amplification-based SPR biosensor showed promising potential for clinical application.

Although the strength of SPR lies in its ability to facilitate reagent-less detection, people often aim to apply different approaches to amplify the signal observed for extremely low concentrations of targets. It is worth mentioning that apart from the relevant LOD achieved in detection, the characteristics of the developed SPR biosensor, such as its high specificity and good precision, satisfy the clinical requirements for the detection of AFP, CEA, and CYFRA 21-1. It is likewise expected that antibody-QD conjugates-based signal amplification would also have utility in mass-sensitive measurements, such as in a quartz crystal microbalance (QCM) biosensor. Further, the specificity of the biosensor may be improved by using aptamers instead of antibodies, which should be investigated in future study. Interestingly, it can be expected that the SPR biosensor can be readily applied for the detection of other tumor markers as well. If so, the approach presented in this work can be used to extend the application of the SPR biosensor for cancer screening in large-scale populations as well.

## Methods

### Chemicals and Materials

QDs (CdSe/ZnS core/shell structure, ~10 nm diameter) at an initial concentration of 1 μM were purchased from Wuhan Jiayuan Quantum Dots Co. Ltd. (Wuhan, China). AuNPs with an average diameter of 15 nm were purchased from JY Biotech (Shanghai, China).

Standard grade of AFP, CEA and CYFRA 21-1; primary (monoclonal, mouse) anti-AFP_1_, anti-CEA_1_ and anti-CYFRA 21-1_1_ (Ab_1_), secondary (monoclonal, mouse) anti-AFP_2_, anti-CEA_2_ and anti-CYFRA 21-1_2_ (Ab_2_) antibodies were purchased from Key-Bio Co. Ltd. (Beijing, China).

HDT, 2,2′-dithiobis[1-(2-bromo-2-methylpropionyloxy)]ethane (DTBE), N,N-dimethylformamide (DMF), aminohexanethiol (AHT), ethyl-3-(3-dimethylaminopropyl) carbodiimide hydrochloride (EDC), and bovine serum albumin (BSA) were purchased from Sigma Aldrich (St. Louis, MO, USA). Human serum albumin (HSA) and human immunoglobulinG (IgG) standards were obtained from Linc-Bio Science Co. Ltd. (Shanghai, China). The buffer used in this work was 0.01 M phosphate-buffered saline (PBS, pH 7.4). All the solutions were prepared with deionized water from a Millipore Milli-Q system.

### SPR biosensor

The SPR biosensor modified in our laboratory, including a polarized light source, sample-loading chamber, micro-flow pump, temperature control system and eight-channel series detection groove, was built by Cyto Trend Biotech Engineering (Beijing, China). The SPR biosensor has the capacity to perform eight tests simultaneously in a single measurement and uses back and forth flow for minimizing the amount of sample used. Glass prisms coated with 50 nm-thick gold film were obtained from GP Medical Technology (Beijing, China).

### Preparation of the SPR chip

A self-assembled monolayer of HDT was developed by immersing a clean Au-coated chip of the glass prism into freshly prepared 2 mM of HDT ethanol solution for 24 h at room temperature. After thoroughly rinsing with ethanol and deionized water, the HDT-modified Au chip was exposed to 0.1 μM of colloidal AuNP solution (diluted using deionized water) for 30 min in darkness at room temperature to fabricate the AuNP monolayer on the surface of the chip^50^. A field emission scanning electron microscope (LEO SUPRA 35; Oberkochen, Germany) was used to analyze the surface morphology to confirm the uniform distribution of AuNPs over the chip surface.

### Preparation of AuNP@Ab_1_ conjugates

First, the self-assembled monolayer (SAM) of AHT was immobilized on the prepared AuNP-coated chip by immersing the chip in 3.2 mM AHT ethanolic solution for 30 min at room temperature. The modified chips were then thoroughly rinsed with 0.01 M PBS (pH 7.4), and Ab_1_ molecules (0.5 mg/mL anti-AFP_1_, 0.6 mg/mL anti-CEA_1_, and 0.65 mg/mL anti-CYFRA 21-1_1_) with carboxylic groups activated by EDC (0.4 mg/mL, 30 min) were attached to three parallel detection channels of the modified chip via amide bonds to AHT-SAM. After the Ab_1_-binding step, the detection channels were completely rinsed with buffer. Finally, the prepared chip was incubated in 1% BSA solution for 30 min to block nonspecific sites.

For specificity detection, some chips were only coated with anti-AFP_1_ by covalent immobilization of anti-AFP_1_ onto the -COOH surface of the chip in the absence of AuNPs.

### Preparation of Ab_2_@QD conjugates

First, 20 μL DTBE solution (0.1% [v/v] in DMF) was slowly added into 1 mL citrate-capped QD solution and gently stirred for 1 h. To prepare the Ab_2_@QD conjugates, 500 μL QD-DTBE solution was slowly added into 10.0 mL of the Ab_2_ solutions (0.1 mg/mL in 0.01 M PBS buffer). The mixture solution was incubated at 37 °C for 1 h and then centrifuged at 12,000 rpm for 10 min three times. Finally, the Ab_2_@QD conjugates were dispersed in PBS buffer with 0.1% BSA.

### SPR real-time detection

The system was maintained at 25 °C, and the PBS buffer was piped into the detection cell at a speed of 100 μL/min for 15 min to equilibrate the chip surface. To analyze the samples, 20 μL each of the AFP, CEA, and CYFRA 21-1 solutions of different concentrations were piped into the corresponding detection channels and circulated for 30 min at a speed of 50 μL/min to allow the reaction to complete, which allowed the targets to combine with the corresponding AuNP@Ab_1_ conjugates on the chip surface. After completion of the reaction, the chip surface was washed and equilibrated with PBS buffer for about 15 min at a speed of 100 μL/min. The Ab_2_@QD conjugates were then piped into each detection channel at 50 μL/min for 30 min separately. The resulting solution was then washed away by pumping PBS buffer at the same flow rate.

### Electrochemiluminescence assay

An automatic electrochemiluminescence immunoassay system was used for the detection of AFP, CEA, and CYFRA 21-1 using the ratio of sample signal to cutoff signal (E170, Roche, Sweden). The automatic immunoassay system was performed using different reagents for determination of the serum concentrations of AFP, CEA, and CYFRA 21-1 (Roche Diagnostics, Mannheim, Germany). Human serum samples were provided by the Southwest Hospital of the Third Military Medical University.

## Additional Information

**How to cite this article**: Wang, H. *et al.* A SPR biosensor based on signal amplification using antibody-QD conjugates for quantitative determination of multiple tumor markers. *Sci. Rep.* **6**, 33140; doi: 10.1038/srep33140 (2016).

## Supplementary Material

Supplementary Information

## Figures and Tables

**Table 1:** Recovery tests of AFP, CEA, and CYFRA 21-1 in human serum by the SPR biosensor.

	Content (ng/mL)	Increment (ng/mL)	Total content^a^ (mean ± SD, ng/mL)	Recovery (%)
AFP	7.3	10	17.5 ± 1.5	101.2
	9.9	10	21.4 ± 1.9	107.5
	89.4	50	137.6 ± 13.2	98.7
	203	100	288.5 ± 27.8	95.2
	218	100	306.9 ± 30.3	96.5
CEA	31.5	10	38.7 ± 3.2	93.2
	18.7	10	32.1 ± 2.6	111.8
	45.6	50	107.2 ± 9.4	112.1
	52.1	100	163.4 ± 12.3	107.4
	126	100	252.1 ± 21.5	111.5
CYFRA 21-1	6.8	10	17.5 ± 1.4	104.2
	12.4	10	21.3 ± 2.1	95.1
	22.7	50	78.7 ± 8.1	108.3
	32.6	50	81.9 ± 7.8	99.2
	204	100	312.4 ± 29.7	102.8

^a^Three replicates per specimen were measured.

SD means standard deviation.

**Table 2:** Comparison of SPR biosensor and electrochemiluminescence method for detection of AFP, CEA, and CYFRA 21-1.

sample	Electrochemiluminescence			SPR biosensor			Relative error (%)		
	AFP	CEA	CYFRA21-1	AFP	CEA	CYFRA21-1	AFP	CEA	CYFRA21-1
1	8.6	5.2	6.8	7.8	5.5	6.3	9.3%	5.8%	7.3%
2	264	6.6	7.3	247	3.1	6.7	6.4%	9.0%	8.2%
3	774	23.4	6.7	813	21.2	6.2	5.0%	9.4%	7.5%
4	7.4	583	7.4	6.8	632	8.1	8.1%	8.4%	9.5%
5	4.8	126	6.4	3.4	137	5.8	8.3%	8.7%	9.4%
6	4.2	6.7	69.7	3.8	6.1	73.8	9.5%	8.9%	5.9%
7	12.1	9.2	257	10.9	10.1	274	9.9%	9.8%	6.6%
8	341	5.9	11.8	365	6.4	12.7	7.0%	8.5%	7.6%
9	15.7	37.5	458	14.3	41.2	499	8.9%	9.8%	9.0%
10	4.3	3.6	5.1	2.0	3.3	4.6	9.3%	8.3%	9.8%
