# Supplementary material for: A SPR biosensor based on signal amplification using antibody-QD conjugates for quantitative determination of multiple tumor markers
Source: Sci Rep. 2016 Sep 12;6:33140. doi: 10.1038/srep33140 (PMC5018874; doi:10.1038/srep33140)
Supplement: Supplementary Information [file srep33140-s1.pdf]

A SPR biosensor based on signal amplification using antibody-QD conjugates for quantitative determination of multiple tumor markers

Huan Wang [1, 3, +], Xiaomei Wang [2, +], Jue Wang [1], Weiling Fu [1], Chunyan Yao [1, *]

[1] Department of Laboratory Medicine, Southwest Hospital, the Third Military Medical University, Chongqing 400038, China

[2] Department of Geriatrics, Southwest Hospital, the Third Military Medical University, Chongqing 400038, China

[3] Chongqing Communication Institute, Chongqing 400035, China

Correspondence and requests for materials should be addressed to C.Yao (email: yao_yao24@yahoo.com)

+ These authors contributed equally to this work.

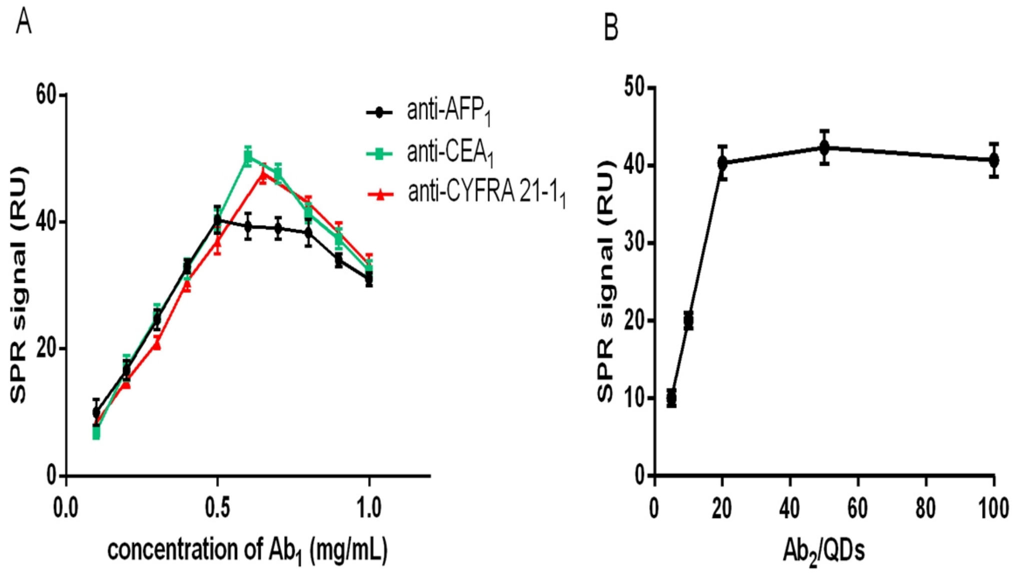

Supplementary Figure 1. Optimization of experimental parameters. (**A**) Effect of different concentrations of anti-AFP_1, anti-CEA_1, and anti-CYFRA 21-1_1 on the SPR detection signal. The assay was performed using 100 ng/mL AFP, CEA, and CYFRA 21-1. (**B**) Effect of different ratios of Ab_2/QDs on the SPR detection signal. The assay was performed using 0.1 mg/mL anti-AFP_2 and 1 μM QDs.

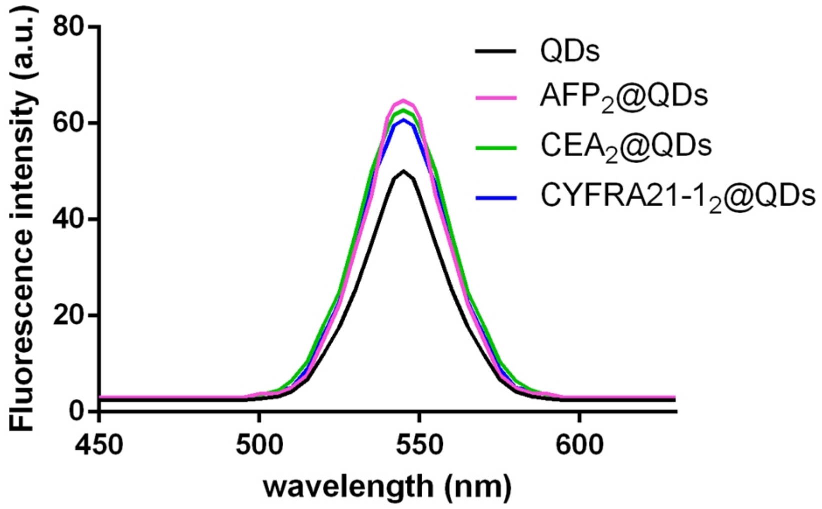

Supplementary Figure 2. Typical fluorescence spectra of QDs before and after coupling with Ab_2.

target	method	Detection limit (ng/mL)	Linear range (ng/mL)	References
AFP	Immunochromatographic test strip	3	3-120	14
	Fluorescence polarization immunoassay	0.28	0.5-500	9
	Carbon-gold nanocomposite	3.1	0.01-100	13
	immunosensor	0.5	1-90	18
	SPR imaging	5.0	5.0-100	40
	SPR biosensor	0.1	0.1-1000	proposed method
CEA	Immunochromatographic test strip	2	2-150	14
	Fluorescence polarization immunoassay	0.36	0.5-500	9
	Carbon-gold nanocomposite	2.7	0.01-100	13
	immunosensor	0.2	0.5-45	18
	SPR biosensor	0.1	0.1-1000	proposed method
CYFRA21-1	FET biosensor	1.0	1.0-1000	17
	SPR biosensor	0.1	0.1-1000	proposed method

SPR: surface plasmon resonance; FET: field effect transistor.

Supplementary Table 1. The results of comparing with other methods.
